# Fluorescent Microangiography Is a Novel and Widely Applicable Technique for Delineating the Renal Microvasculature

**DOI:** 10.1371/journal.pone.0024695

**Published:** 2011-10-03

**Authors:** Andrew Advani, Kim A. Connelly, Darren A. Yuen, Yanling Zhang, Suzanne L. Advani, Judy Trogadis, M. Golam Kabir, Etai Shachar, Michael A. Kuliszewski, Howard Leong-Poi, Duncan J. Stewart, Richard E. Gilbert

**Affiliations:** 1 Keenan Research Centre of the Li Ka Shing Knowledge Institute, St. Michael's Hospital, Toronto, Ontario, Canada; 2 Ottawa Hospital Research Institute, University of Ottawa, Ottawa, Ontario, Canada; University of Colorado Denver, United States of America

## Abstract

Rarefaction of the renal microvasculature correlates with declining kidney function. However, current technologies commonly used for its evaluation are limited by their reliance on endothelial cell antigen expression and assessment in two dimensions. We set out to establish a widely applicable and unbiased optical sectioning method to enable three dimensional imaging and reconstruction of the renal microvessels based on their luminal filling. The kidneys of subtotally nephrectomized (SNx) rats and their sham-operated counterparts were subjected to either routine two-dimensional immunohistochemistry or the novel technique of fluorescent microangiography (FMA). The latter was achieved by perfusion of the kidney with an agarose suspension of fluorescent polystyrene microspheres followed by optical sectioning of 200 µm thick cross-sections using a confocal microscope. The fluorescent microangiography method enabled the three-dimensional reconstruction of virtual microvascular casts and confirmed a reduction in both glomerular and peritubular capillary density in the kidneys of SNx rats, despite an overall increase in glomerular volume. FMA is an uncomplicated technique for evaluating the renal microvasculature that circumvents many of the limitations imposed by conventional analysis of two-dimensional tissue sections.

## Introduction

Glomerular and peritubular capillary loss are major histopathological features of most forms of chronic kidney disease (CKD) that, by predisposing to tissue hypoxia, are also fundamentally linked to its progression [1]. Accordingly, the ability to attenuate further microvascular loss and assist with its regeneration is now viewed as an important contributor to the effects of renoprotective therapies [2], [3]. Unfortunately, laboratory methods for the detailed assessment of the kidney microvasculature are overly labor intensive and the vast majority of studies exploring the therapeutic efficacy of novel agents rely on conventional light microscopic examination of antibody- or lectin-stained kidney sections. Such techniques, while simple to perform, are clearly limited not only by their assessment in two dimensions [3], but also by the lack of specificity of the labeling techniques employed [4]. To address the need for a widely applicable approach for delineating the renal microvasculature in three dimensions, we developed the novel technique of fluorescent microangiography (FMA).

The FMA technique relies on low-temperature melting point agarose with fluorescent polystyrene microspheres to provide a microangiogram that can be examined by confocal microscopy and that can be readily analyzed using both free and commercial software. In the present study, the utility of this approach for assessing the microvascular architecture of the kidney in the physiological and disease settings was assessed in sham-operated and subtotally nephrectomized (SNx) rats, respectively.

## Results

**Figure 1:**
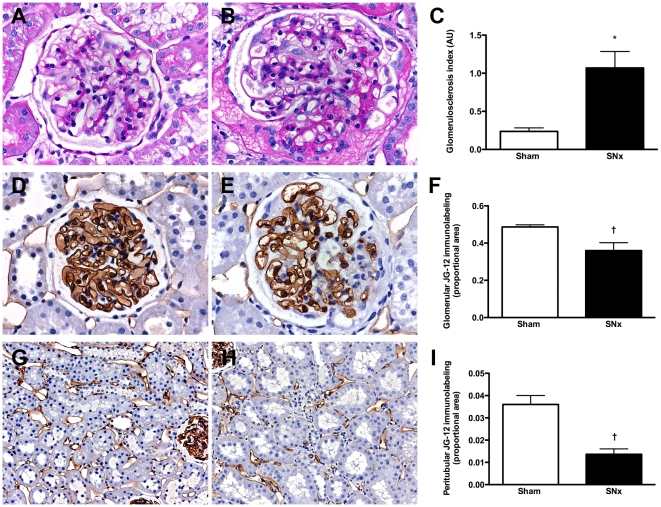
Renal structure in sham and subtotally nephrectomized (SNx) rats. (A and B) PAS-stained kidney sections from sham (A) and SNx (B) rats. Original magnification ×400. (C) Glomerulosclerosis index. (D and E) Glomerular JG-12 immunolabeling from sham (D) and SNx (E) rats. Original magnification ×400. (F) Quantitation of glomerular JG-12 immunostaining. (G and H) Peritubular JG-12 immunolabeling from sham (G) and SNx (H) rats. Original magnification ×160. (I) Quantitation of tubulointerstitial JG-12 immunostaining. *p<0.001, ^†^p<0.05.

To evaluate the utility of kidney FMA we employed an established model of progressive, proteinuric kidney disease, the SNx rat, in which the pathological aberration of the renal microvasculature has been previously well described [5], [6]. In comparison to sham-operated animals, SNx rats demonstrated an increase in systolic blood pressure (SBP) and urine protein excretion with a decline in glomerular filtration rate (GFR) (Table 1). Light microscopic survey of kidney sections from SNx rats revealed the presence of glomerular enlargement, focal inflammatory cell infiltrates with dilated, atrophic tubules and increased deposition of extracellular matrix within both the glomerular and tubulointerstitial compartments. The magnitude of glomerular injury was estimated semi-quantitatively on PAS-stained kidney sections and, as expected, was increased in SNx rats in comparison to sham animals (Figure 1A–C). To examine the utility of the FMA approach in delineating the renal microvasculature in sham and SNx rats, capillary density was first determined in conventional 4 µm-thick formalin-fixed, paraffin-embedded kidney sections. To avoid the confounding effect of lymphatic labeling, kidney sections were immunostained with a monoclonal antibody (JG-12) that binds endothelial cells of capillaries, but not lymphatics in rat kidney [4]. With the assistance of image analysis software, the proportional area of endothelial immunostaining was observed to be reduced within both the glomeruli (Figure 1D–F) and the cortical tubulointerstitium (Figure 1G–I) of SNx rats in comparison to their sham-operated counterparts.

**Table 1:** Functional characteristics of sham and subtotally nephrectomized (SNx) rats.

	Sham	SNx
Body weight (g)	682±27	638±16
Kidney weight (g)	2.17±0.15	2.45±0.20
Kidney weight, body weight (%)	0.31±0.01	0.38±0.03
SBP (mmHg)	134±8	182±8*
Proteinuria (mg/day)	18.28×/÷1.18	61.24×/÷1.45†
Urine protein∶creatinine ratio (mg/µmol)	0.10×/÷1.18	0.39×/÷1.44†
GFR (ml/min/kg)	7.71±0.57	1.74±0.43‡

*p<0.01,

† p<0.05,

‡ p<0.001.

**Figure 2:**
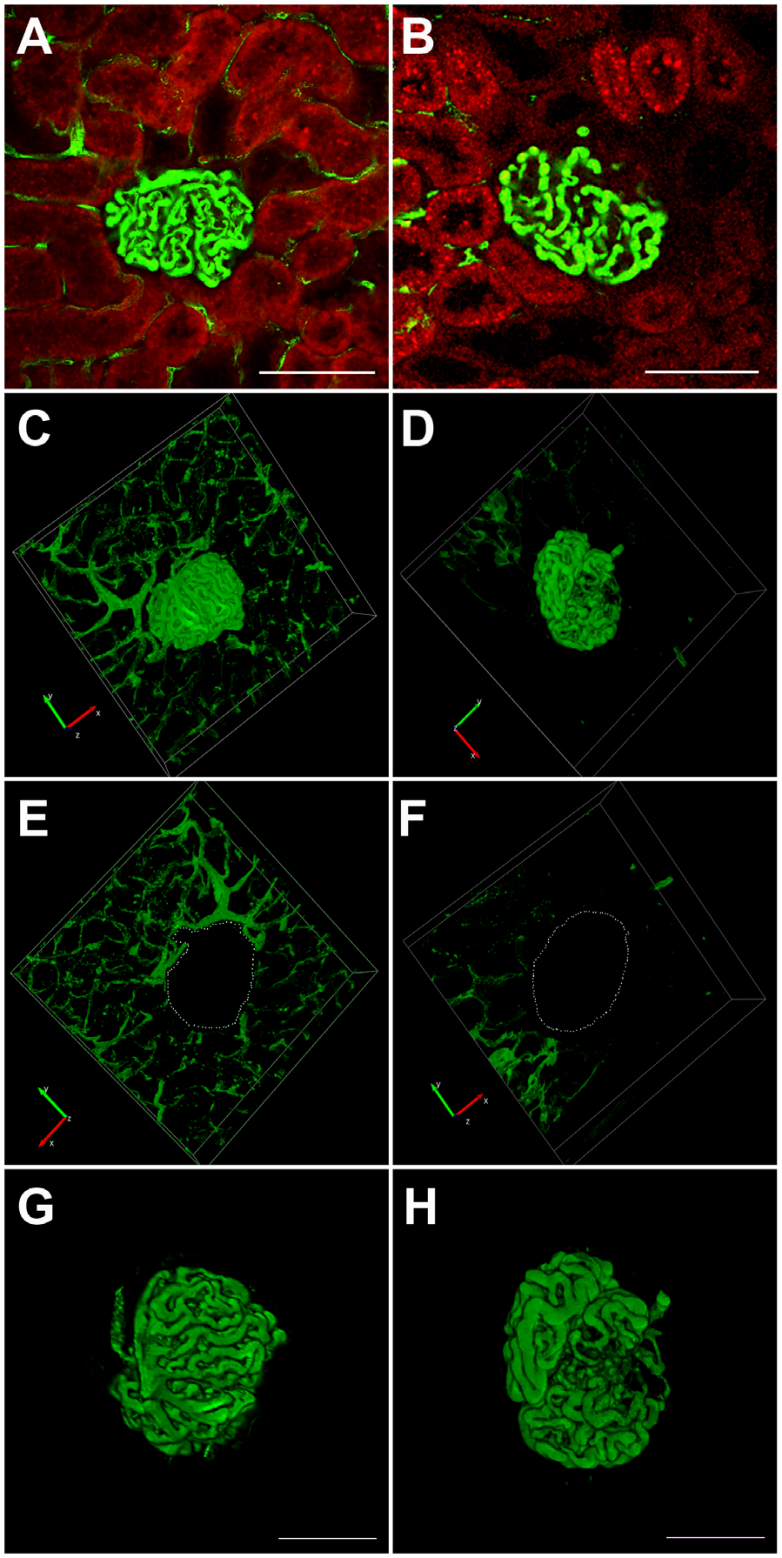
Fluorescent microangiography (FMA) in kidney sections from sham (A, C, E, G) and subtotally nephrectomized (SNx) rats (B, D, F, H). Figures 2A and 2B are single optical sections of rat kidneys perfused with the agarose/fluorescent microsphere mixture (green), red colour is tubular autofluorescence. Figures 2C–2H are reconstructions of the same perfused glomeruli shown in A and B using Neurolucida software. For .avi movies of the three-dimensional reconstructions shown in C and D please see the online supplement (Movie S1 and S2). Figures 2E and F and Figures 2G and H show how the technique can be used for delineating the peritubular (E and F) and glomerular (G and H) capillaries independently. Scale bar = 100 µm.

**Figure 3:**
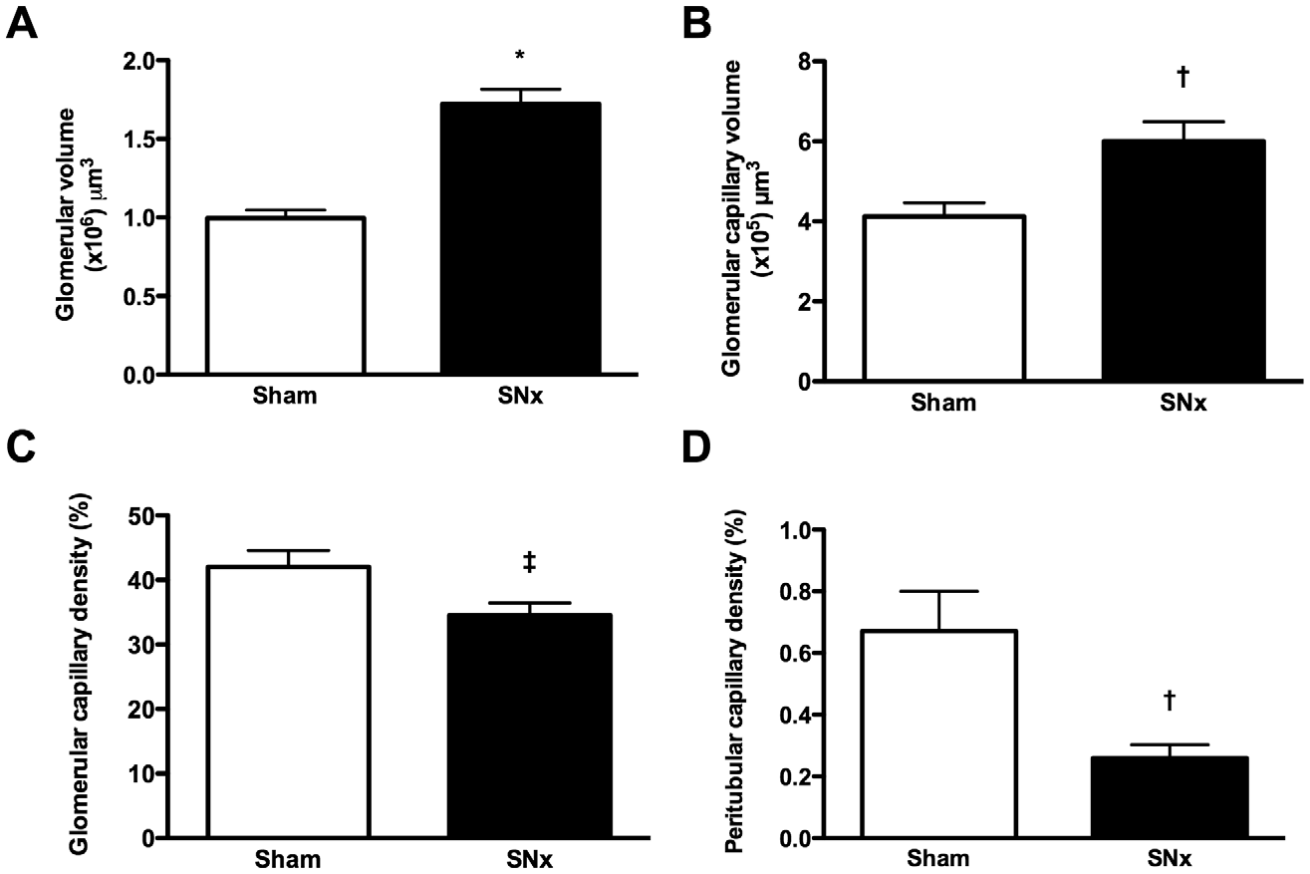
Microvascular parameters in sham and subtotally nephrectomized (SNx) rats determined by fluorescent microangiography (FMA). (A) Glomerular volume. (B) Glomerular capillary volume. (C) Glomerular capillary density. (D) Peritubular capillary density. *p<0.0001, ^†^p<0.01, ^‡^p<0.05.

A range of pilot and optimization experiments identified that visualization of the glomerular and peritubular capillaries, with the FMA method, was optimally achieved by perfusing the kidney, at systolic pressure, with a pre-warmed 1% low melting point agarose solution containing 10% 0.02 µm fluorescent microspheres and preceded by the delivery, via the renal artery, of heparin (100 IU) and 3 M KCl. Following fixation, thick kidney cross-sections were obtained with a vibrating microtome blade that, after mounting, enabled the direct determination of microsphere fluorescence across the confocal z-stack. Two software packages were employed for either the reconstruction of three-dimensional images of the glomerular and peritubular capillary network (Neurolucida, MBF Bioscience, Williston, VT) (Figure 2) or determination of capillary volume (ImageJ, NIH, Bethesda, MD) (Figure 3).

Figure 2 illustrates single optical sections from either sham-operated or SNx rats (Figure 2A and B) and the serial progression to the creation of three-dimensional reconstructions of either the glomerular or peritubular capillary network (Figure 2C–H). The online supplement shows .avi movies of the reconstructed glomeruli (Movie S1 and S2). Using this technique, we observed that while glomerular capillary volume was increased in SNx rats, glomerular capillary density was reduced, by virtue of both an increase in overall glomerular volume and by the segmental accumulation of mesangial matrix, reflected by segmental capillary dropout (Figure 3A–C). Similarly, within the cortical tubulointerstitium, peritubular capillary density was also reduced in SNx rats (Figure 3D).

## Discussion

The FMA method, described herein, combines several readily available technologies that enable its potential broad application. These include an agarose mixture with viscosity similar to blood within microvessels [7], fluorescent microspheres that avoid the confounding effects of dye sequestration, the optical sectioning capabilities of modern confocal microscopy and computer software that can accurately generate three dimensional reconstructions (Neurolucida). Beyond this, the application of a pixel counting software (ImageJ), freely available for download from the National Institutes of Health, enables the calculation of numerical dimensions from the acquired optical sections in a fraction of the time required for conventional morphometric analyses of serial images.

Traditionally, assessment of the renal vasculature has been achieved using a range of antibodies directed against endothelial cell surface antigens. While generally reliable, such an immunohistochemical strategy relies on several assumptions. For instance, it is usually assumed that surface antigens are evenly distributed on luminal and/or abluminal membranes in all tissues and that the labeling is not only specific for the blood vessel endothelium but that the extent of cell surface expression remains relatively stable over time and with disease. Detailed examination has, however, shown that this is not the case. Indeed, several antibodies directed against the blood vessel endothelium, such as CD31 (*Platelet Endothelial Cell Adhesion Molecule*, PECAM-1), CD34 and von Willebrand factor, also target cells of hematopoietic origin as well as lymphatic endothelia, consistent with the common embryological derivation of these cell types. Differentiating blood and lymphatic endothelia may be particularly important in the setting of tubulointerstitial fibrosis where both an increase and diminution of capillary density have previously been reported in the same disease model, the SNx rat [4], [8]. To delineate blood and lymphatic endothelia, Kim and colleagues previously employed a monoclonal antibody (JG-12), directed against aminopeptidase P, that had been shown to intensely label blood but not lymphatic vessel endothelium in the kidney and lung [9]. These studies demonstrated a loss of blood vessel endothelium in conjunction with lymphangiogenesis in the SNx rat [4]. However, while ostensibly specific for blood vessel endothelium, aminopeptidase P itself is also found on brush-border cells in the intestine and lymphoid cells [10]. Moreover, its expression may be modified by the surrounding milieu, as has been observed in the lung adenocarcinoma setting for example [11]. By assessing blood vessel lumina only, FMA avoids being confounded by the subtleties of shifting endothelial antigens and their co-expression by the lymphatic vasculature.

In addition to examining the normal kidney, we also used FMA to explore microvasculature changes in the disease setting. Like light microscopy, FMA revealed a decrease in glomerular capillary density, along with an increase in capillary volume in SNx rats. As previously reported [5], loss of peritubular capillaries was even more dramatic than those of the glomerulus after subtotal nephrectomy. While reports, by and large, have historically focused on changes within the glomerular endothelium, more recent studies have revealed that disparate changes may, under some circumstances, be observed within the distally located peritubular capillaries [12]. Thus, an experimental approach that facilitates the ready appreciation of both vascular compartments within the kidney is clearly advantageous. The complexity of the renal microvasculature is evidenced, not only in the renal glomerulus, but also by much of the cortical nephron that is perfused by the highly anastamotic intertubular plexus. Accordingly, tracking the course of an individual capillary in a two dimensional tissue section is virtually impossible. Indeed, even in the medulla where capillaries follow the loops of Henle, the likelihood that the plane of sectioning might match that of an individual *vas rectum* is extraordinarily low.

A range of methods have previously been used for the three dimensional reconstruction of the microvasculature, including the infusion of barium sulfate–gelatin. However, because of its high viscosity, this technique requires high perfusion pressures that lead to distention of the proximal vasculature thereby limiting its utility for microvascular study [7]. In contrast, the viscosity of the agarose gel used in the present study is <5 centipoise at 37°C [7], similar to that of capillary blood [13], [14]. Other methods that have been employed to provide three dimensional images of the microvasculature include corrosion casting [15] and CT microangiography [16] that, unlike FMA, are limited by their destruction of surrounding tissue or requirement for highly specialized equipment, not available in most laboratories. The mean glomerular capillary volume of 4.1×10^5^ µm^3^, determined by FMA in sham-operated rats in the present study, is consistent with previously reported rat glomerular capillary volumes of 2–5×10^5^ µm^3^ determined by gold standard morphometric techniques following transmission electron microscopy [17], [18]. Accordingly, although it remains plausible that cooling of the agarose perfusate may result in vessel dilatation we think this effect is likely to be minimal and would not preclude between group comparisons.

During optimization of the FMA method we identified a number of nuances that facilitated optimal delineation of the renal microvessels. Uniform filling of the vessels was critical for accurate software analysis to establish connectivity. A variety of attributes of the agarose/bead mixture affected filling including a tendency for clumping of the fluorescent beads, the size of the beads and their concentration in the agarose solution. To reduce clumping, the original bead solution was briefly vortexed immediately prior to being added to the agarose solution. Initially, we used 0.2 µm size beads but later reduced this to 0.02 µm for more even distribution in the agarose. A lower concentration resulted in less even filling. The perfusion conditions of the animal also appeared critical. Firstly, heparinization prevented coagulation artefacts from impeding luminal filling. Secondly, infusion pressures were maintained at physiologic systolic levels for both sham and SNx rats, with KCl administered to induce vasodilatation [19]. Thirdly, optimal warming and cooling of the agarose mixture was pivotal. In this regard, the low-melting point agarose mixture was warmed to just above 40°C and the organ perfused with saline that was at room or body temperature. Immediately after perfusion the kidney was cooled with ice before transfer of the whole animal to ice for 10 minutes. With respect to visualization of the beads by confocal microscopy, the intense fluorescence enabled deep tissue penetration (10-fold greater than FITC [7]) and, after trialing a variety of mounting media, we found 2,2′-thiodiethanol (TDE) optimally enabled us to image through the thick specimens. TDE is a water soluble mounting medium that clears tissue, decreases light scattering, minimizes refractive index changes from within deep tissue and improves axial resolution [20].

In summary, FMA is a novel technique that facilitates the visualization and quantification of the renal microvasculature and circumvents many of the limitations afforded by conventional analyses of individual and serial two-dimensional kidney sections. The simplicity and wide applicability of the technique should facilitate its adoption for the assessment of renal vascular injury in an array of disease models. It does not, however, supplant the need for rigorous methods for quantitative analysis [21], [22], though the potential combination of FMA with computer-assisted quantitative algorithms that incorporate the complex topography of the renal microvascular network [23] suggest this may, with time, become less onerous.

## Materials and Methods

### Ethics statement

All animal work was conducted according to the Canadian Council on Animal Care Guidelines. The specific experimental protocol, ACC 983, was approved by the Animal Care Committee of St. Michael's Hospital.

### Animals

Male Sprague Dawley rats (Charles River, Montreal, Quebec) weighing 563±8 g were randomized to undergo either subtotal nephrectomy (SNx, n = 14) or sham surgery (n = 7) as previously described [24]. Briefly, animals were anaesthetized with 2.5% inhaled isoflurane, the right kidney was removed via subcapsular nephrectomy and infarction of approximately two thirds of the left kidney was achieved via selective ligation of two out of the three or four branches of the left renal artery. Sham surgery consisted of laparotomy and manipulation of both kidneys before wound closure. Rats were maintained for 12 weeks post-surgery.

After 12 weeks, assessments were made of GFR, urine protein excretion and SBP. GFR was determined by FITC-inulin clearance, using an adaptation of the protocol recommended by the Animal Models of Diabetes Complications Consortium (AMDCC), available at http://www.amdcc.org/shared/showFile.aspx?doctypeid=3&docid=28, with FITC-inulin delivery and repeated sampling achieved via the tail-vein. Urine protein excretion and protein∶creatinine ratio were determined after housing rats individually in metabolic cages for 24 h with subsequent measurement of urine protein with the benzethonium chloride method and urine creatinine with the Jaffé method. SBP was determined in conscious rats using an occlusive tail-cuff plethysmograph attached to a pneumatic pulse transducer (Powerlab, ADInstruments, Colorado Springs, CO), as previously described [25].

At sacrifice, kidney tissue was collected in the following manner: for SNx rats, the remnant kidney was either fixed in 10% neutral buffered formalin (NBF) (immersion-fixation, n = 7; perfusion-fixation, n = 2) for routine histological analysis or subjected to fluorescent microangiography (FMA) (see below, n = 5). For sham rats, the right kidney was fixed in 10% NBF (immersion-fixation, n = 5; perfusion-fixation, n = 2) and the left kidney was subjected to FMA (see below, n = 5).

### Glomerulosclerosis index

Eighty glomeruli were examined in PAS-stained kidney sections from each rat. The degree of sclerosis was subjectively graded on a scale of 0 to 4 as previously described [26]: grade 0, normal; grade 1, sclerotic area up to 25% (minimal); grade 2, sclerotic area 25% to 50% (moderate); grade 3, sclerotic area 50% to 75% (moderate to severe); and grade 4, sclerotic area 75% to 100% (severe). Glomerulosclerosis was defined as basement membrane thickening, mesangial hypertrophy and capillary occlusion. A glomerulosclerosis index (GSI) was then calculated using the formula:
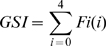



Where Fi is the percentage of glomeruli in the rat kidney with a given score (i).

### Immunohistochemistry and conventional determination of capillary density on two-dimensional kidney sections

Glomerular endothelial cells were recognized using anti-rat epithelial aminopeptidase P monoclonal antibody JG-12 (Bender Medsystems GbdH, Vienna, Austria) which binds to endothelial cells of blood vessels, but not to lymphatics in rat kidney [4]. Immunohistochemistry was performed as previously described [27], [28], with omission of primary antisera serving as the negative control. Kidney sections were scanned with the Aperio ScanScope system (Aperio Technologies Inc., Vista, CA) and analysed using ImageScope (Aperio). For determination of glomerular capillary density, the proportional glomerular area of JG-12 immunostaining was determined in 30 randomly selected glomerular profiles from each kidney section. For estimation of peritubular capillary density, the proportional area of JG-12 immunostaining (excluding glomeruli) was determined in 10 randomly selected cortical fields (×100 magnification).

### Fluorescent microangiography

Rats were anaesthetized with 2% isoflurane in a supine position. The abdomen was opened through a midline incision extending from the xiphoid process to the symphysis pubis and the abdominal aorta dissected from the level of the superior mesenteric artery to its bifurcation. The abdominal aorta was then ligated proximal to the renal artery and distally at the level of the aortic bifurcation and 1 ml of heparinized saline (100 IU/ml heparin, 0.9% NaCl), followed by 1 ml of 3 M KCl were delivered via a 30G needle. Immediately after heparinization, the kidney was perfused, at systolic pressure, with 100 ml 0.9% saline using an 18G angiocath with perfusion-exsanguination facilitated by severance of the external jugular vein. The pre-warmed (40°C) agarose-fluorescent microbead mixture (1% low melting point agarose (Sigma) and 10% 0.02 µm fluospheres (Invitrogen, Carlsbad, CA)) was then delivered via the angiocath. After infusion, the rat was cooled on ice for 10 min and the kidney was removed and immersion fixed in 10% NBF for 48 h at 4°C.

After fixation, 200 µm thick kidney cross-sections were obtained using a vibrating-blade microtome (VT100SR, Leica, Nussloch, Germany) with a cutting speed of 0.025 mm/s and vibration frequency of 100 Hz. Sections were washed in PBS overnight and incubated in serial concentrations of water soluble embedding medium (2,2′-thiodiethanol (TDE, Sigma)), before mounting in 95% TDE.

Serial images were collected with a confocal microscope (Leica TCS SL, Leica, Richmond Hill, ON; St. Michael's Hospital Bioimaging Facility) (20× objective, zoom 2, NA 0.75) across the z-stack (0.8141 µm steps) and the fluorescent microspheres were viewed with an argon laser (excitation 488 nm, emission 515 nm). Six glomeruli were evaluated from each animal. Glomerular capillary volume was calculated by pixel counting using ImageJ version 1.39 (from the National Institutes of Health and available at http://rsb.info.nih.gov/ij/). Peritubular capillary volume was estimated from the same optical sections with the glomerular capillary tuft excluded with the aid of the negative pen tool. In each case capillary volume was determined as the product of the positive pixel area in each profile and the distance between each optical image (0.8141 µm). Glomerular volume was determined with ImageJ from the three dimensional reconstructions generated by Neurolucida (MBF Bioscience, Williston, VT), using the formula V = 4πabc/3. Glomerular capillary density was estimated as the quotient of glomerular capillary volume and total glomerular volume.

### Statistics

Data are expressed as means ± SEM except proteinuria which was skew-distributed and is presented as geometric mean ×/÷ tolerance factor. Statistical significance was determined by Student's t-test, with prior log-transformation of skew-distributed proteinuria data. All statistical analyses were performed using GraphPad Prism 5 for Mac OS X (GraphPad Software Inc., San Diego, CA). A p<0.05 was considered statistically significant.

## Supporting Information

Movie S1 **Three-dimensional reconstruction of a glomerulus from a sham-operated rat following fluorescent microangiography (FMA).**
(AVI)

Movie S2 **Three-dimensional reconstruction of a glomerulus from a subtotally nephrectomized (SNx) rat following fluorescent microangiography (FMA).**
(AVI)

## References

[1] Fine LG, Norman JT (2008). Chronic hypoxia as a mechanism of progression of chronic kidney diseases: from hypothesis to novel therapeutics.. Kidney Int.

[2] Fogo AB (2005). New capillary growth: a contributor to regression of sclerosis?. Curr Opin Nephrol Hypertens.

[3] Remuzzi G, Benigni A, Remuzzi A (2006). Mechanisms of progression and regression of renal lesions of chronic nephropathies and diabetes.. J Clin Invest.

[4] Matsui K, Nagy-Bojarsky K, Laakkonen P, Krieger S, Mechtler K (2003). Lymphatic microvessels in the rat remnant kidney model of renal fibrosis: aminopeptidase p and podoplanin are discriminatory markers for endothelial cells of blood and lymphatic vessels.. J Am Soc Nephrol.

[5] Kang DH, Joly AH, Oh SW, Hugo C, Kerjaschki D (2001). Impaired angiogenesis in the remnant kidney model: I. Potential role of vascular endothelial growth factor and thrombospondin-1.. J Am Soc Nephrol.

[6] Kang DH, Kanellis J, Hugo C, Truong L, Anderson S (2002). Role of the microvascular endothelium in progressive renal disease.. J Am Soc Nephrol.

[7] Dutly AE, Kugathasan L, Trogadis JE, Keshavjee SH, Stewart DJ (2006). Fluorescent microangiography (FMA): an improved tool to visualize the pulmonary microvasculature.. Lab Invest.

[8] Pillebout E, Burtin M, Yuan HT, Briand P, Woolf AS (2001). Proliferation and remodeling of the peritubular microcirculation after nephron reduction: association with the progression of renal lesions.. Am J Pathol.

[9] Kim YG, Suga SI, Kang DH, Jefferson JA, Mazzali M (2000). Vascular endothelial growth factor accelerates renal recovery in experimental thrombotic microangiopathy.. Kidney Int.

[10] Lasch J, Moschner S, Sann H, Zellmer S, Koelsch R (1998). Aminopeptidase P–a cell-surface antigen of endothelial and lymphoid cells: catalytic and immuno-histotopical evidences.. Biol Chem.

[11] Oh P, Li Y, Yu J, Durr E, Krasinska KM (2004). Subtractive proteomic mapping of the endothelial surface in lung and solid tumours for tissue-specific therapy.. Nature.

[12] Kosugi T, Nakayama T, Li Q, Chiodo VA, Zhang L (2010). Soluble Flt-1 gene therapy ameliorates albuminuria but accelerates tubulointerstitial injury in diabetic mice.. Am J Physiol Renal Physiol.

[13] Sharman JE, Brown J, Holland DJ, Macdonald G, Kostner K (2009). Influence of altered blood rheology on ventricular-vascular response to exercise.. Hypertension.

[14] Nagaoka T, Yoshida A (2006). Noninvasive evaluation of wall shear stress on retinal microcirculation in humans.. Invest Ophthalmol Vis Sci.

[15] Mondy WL, Cameron D, Timmermans JP, De Clerck N, Sasov A (2009). Micro-CT of corrosion casts for use in the computer-aided design of microvasculature.. Tissue Eng Part C Methods.

[16] Terayama H, Ishikawa M, Yasunaga Y, Yamasaki T, Hamaki T (2011). Prevention of osteonecrosis by intravenous administration of human peripheral blood-derived CD34-positive cells in a rat osteonecrosis model.. J Tissue Eng Regen Med.

[17] Miller PL, Scholey JW, Rennke HG, Meyer TW (1990). Glomerular hypertrophy aggravates epithelial cell injury in nephrotic rats.. J Clin Invest.

[18] Haylor J, Chowdry J, Baillie H, Cope G, el Nahas AM (1996). Renal function and morphometry in the dwarf rat following a reduction in renal mass.. Nephrol Dial Transplant.

[19] Prior HM, Webster N, Quinn K, Beech DJ, Yates MS (1998). K(+)-induced dilation of a small renal artery: no role for inward rectifier K+ channels.. Cardiovasc Res.

[20] Staudt T, Lang MC, Medda R, Engelhardt J, Hell SW (2007). 2,2′-thiodiethanol: a new water soluble mounting medium for high resolution optical microscopy.. Microsc Res Tech.

[21] Bertram JF (1995). Analyzing renal glomeruli with the new stereology.. Int Rev Cytol.

[22] Scruggs BS, Zuo Y, Donnert E, Ma L, Bertram JF (2011). Increased capillary branching contributes to angiotensin type 1 receptor blocker (ARB)-induced regression of sclerosis.. Am J Pathol.

[23] Antiga L, Ene-Iordache B, Remuzzi G, Remuzzi A (2001). Automatic generation of glomerular capillary topological organization.. Microvasc Res.

[24] Yuen DA, Connelly KA, Advani A, Liao C, Kuliszewski MA (2010). Culture-modified bone marrow cells attenuate cardiac and renal injury in a chronic kidney disease rat model via a novel antifibrotic mechanism.. PLoS One.

[25] Gilbert RE, Huang Q, Thai K, Advani SL, Lee K (2011). Histone deacetylase inhibition attenuates diabetes-associated kidney growth: potential role for epigenetic modification of the epidermal growth factor receptor.. Kidney Int.

[26] Advani A, Kelly DJ, Advani SL, Cox AJ, Thai K (2007). Role of VEGF in maintaining renal structure and function under normotensive and hypertensive conditions.. Proc Natl Acad Sci U S A.

[27] Advani A, Kelly DJ, Cox AJ, White KE, Advani SL (2009). The (Pro)renin receptor: site-specific and functional linkage to the vacuolar H+-ATPase in the kidney.. Hypertension.

[28] Advani A, Gilbert RE, Thai K, Gow RM, Langham RG (2009). Expression, localization, and function of the thioredoxin system in diabetic nephropathy.. J Am Soc Nephrol.

